# Supplementary material for: Engineered lactobacilli display anti-biofilm and growth suppressing activities against Pseudomonas aeruginosa
Source: NPJ Biofilms Microbiomes. 2020 Oct 30;6:48. doi: 10.1038/s41522-020-00156-6 (PMC7599214; doi:10.1038/s41522-020-00156-6)
Supplement: Supplementary file 1 — Supplemental File [file 41522_2020_156_MOESM1_ESM.pdf]

Supplemental Information for

Engineered lactobacilli display anti-biofilm and growth suppressing activities against *Pseudomonas aeruginosa*

Todd C. Chappell and Nikhil U. Nair*

Department of Chemical & Biological Engineering, Tufts University

*corresponding author:

4 Colby St, STC 276

Medford, MA 02155

617-627-2582

Email: nikhil.nair@tufts.edu

Twitter: @nair_lab

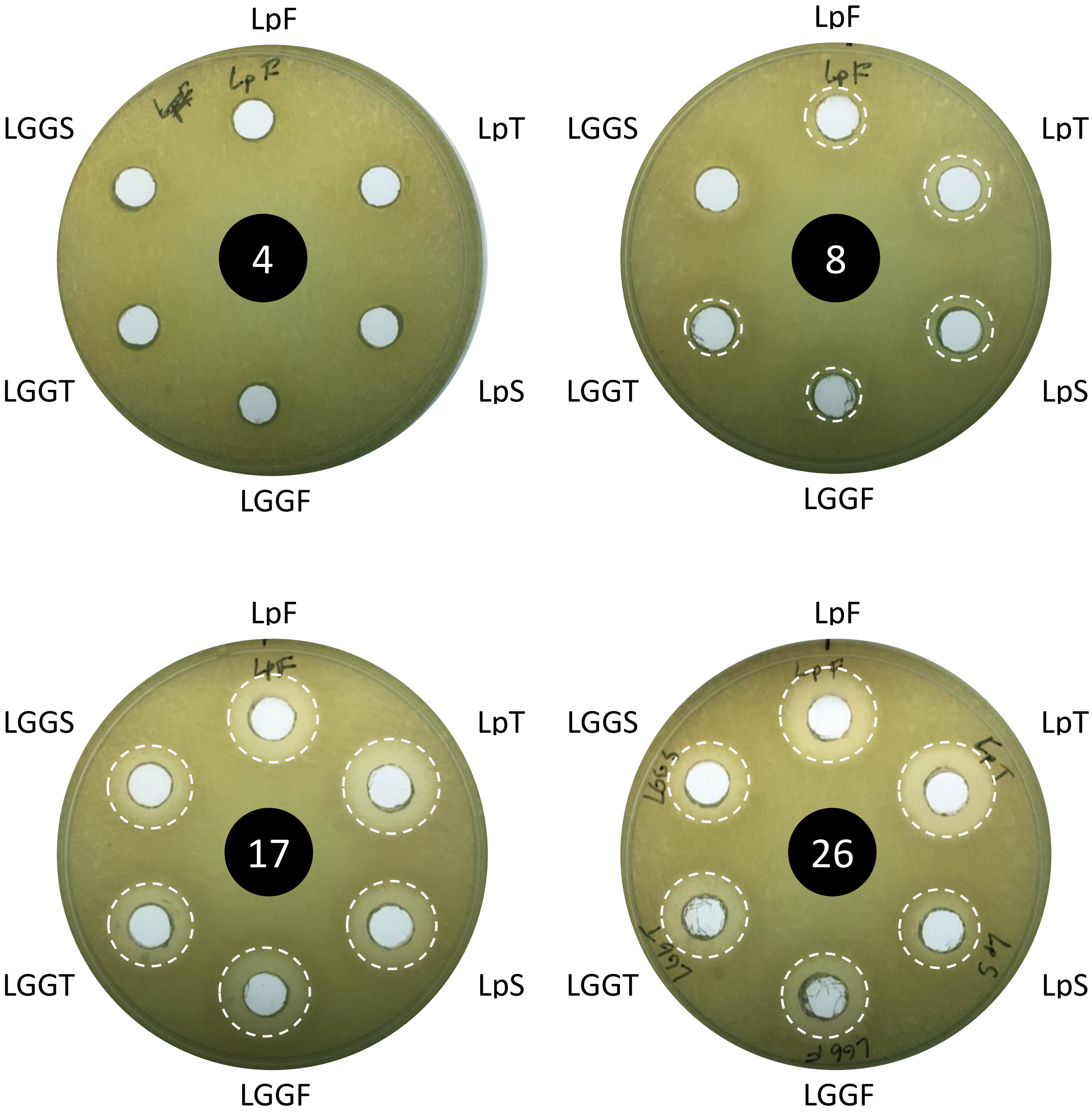

Supplementary Figure 1: Impact of LAB culture aeration and duration on PA14 growth inhibition. *L. plantarum* (Lp) and *L. rhamnosus* (LGG) were grown shaking in a baffled flask (F), shaking in a test tube (T), or statically in a test tube (S) for 4, 8, 17, or 26 hours.

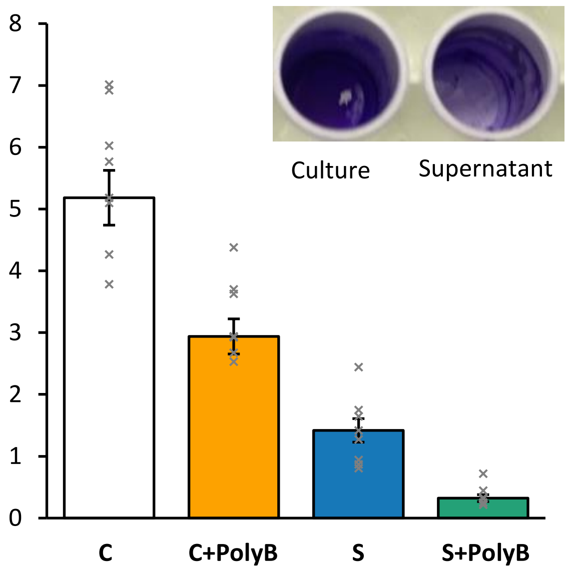

Supplementary Figure 2: Degradation of PA14 biofilms by $PelA_h$ secreting LGG. "C" is LGG cultures and "S" is LGG supernatants. "PolyB" denotes addition of 100 ug/mL polymyxin B. Workflow is the same given in Figure 3. n=9 from 3 separate experiments for each condition. End pH was 6.7 - 7.0. Inset shows crystal violet stained PA14 biofilms after treatment with LGG culture (left) or filtered LGG supernatant (right).

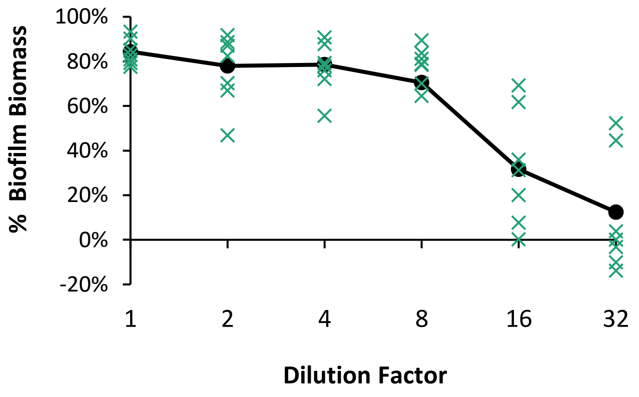

Supplementary Figure 3: PA14 biofilm degradation by diluted *L. plantarum* $PelA_h$ supernatants. *L. plantarum* $PelA_h$ supernatants were diluted in *L. plantarum* control supernatants to determine the range of dilution that still permits PA14 biofilm degradation. Relative biofilm biomass is the average biofilm biomass n=9 from 3 separate experiments for each condition. One outlier was removed from dilution 4 and dilution 32.

Supplementary Table 1: Bacterial strains used in this study.

Strain	Genotype/Use/Origin	Source/Origin
Lactobacillus rhamnosus GG	Human stool isolate [1]	Dr. David R. Snydman, TMC
Lactobacillus plantarum WCFS1	Single colony isolate from NCIMB8826, an isolate of human saliva[2]	Dr. Michiel Kleerebezem, WUR
Pseudomonas aeruginosa PA14	Clinical isolate from burn wound[3]	Dr. Roberto Kolter, HMS
E. coli TG1	cloning	Dr. Ann Hochschild, HMS
E. coli TG1 *endA*⁻	*endA::cat*; cloning	This work
E. coli DH5a Z1	Cloning; R. Lutz and H. Bujard 1997[4]	Dr. Huimin Zhao, UIUC
Staphylococcus aureus UAMS-1	Source of *nucA*.	Dr. Abraham L. Sonenshein, TUSM
Clostridium cellulovorans DSM 3052	Genomic DNA. Source of EngZ	DSMZ

Supplementary Table 2: Plasmids used in this study.

Plasmid	Notes	Source
pLp_3050Ag85B-E6cwa2	Expression vector for *L. plantarum* derived from pSIP401[5]. low copy narrow host 256rep ori + pUC ori. Expression induced by addition of SppIP.	Dr. Geir Mathiesen, NMBU
pSIP411	Broad host, High copy SH71 ori only. LAB expression vector.[6]	Dr. Jan Peter van Pijkeren, UW
pTCC200	pSIP401 with a new multiple cloning site, N-terminal 6x-histidine tag, and Lp_3050 secretion signal	This work
pTCC204	pSIP401 with *engZ*	This work
pTCC210	pSIP411 with a new multiple cloning site, N-terminal 6x-histidine tag, and Lp_3050 secretion signal	This work
pTCC211	pTCC210 with *nucA*	This work
pTCC214	pTCC210 with *engZ*	This work
pTCC216	pTCC210 with $pelA_{hyd}$	This work

Supplementary Table 3: Primers used in this study.

#	Name	Sequence
1	Pro1	gactcagatctaccggtttaatttgaaaattg
2	Pro2	taaaatctccttgtaatagtattttatagaatac
3	Pro3	ctataaaatactattacaaggagattttacatATGAAAAAATTTAACTTTAAAACC
4	Pro4	CCGTGGAACTAAACCTGATGAATGATGATGATGATGATGCGTACGCTTGGAGGCCTGGGC
5	Pro5	gcactcacgtgccatggcatgcgtcgacTGAACCCCGTGGAACTAAACCTGATGAATG
6	nucAF	gactcgtcgactcaactaaaaaattacataaagaacc
7	nucAR	gcactcacgtgttattgacctgaatcagcgttg
8	engZF	gactcgtcgacacagaaaattacaactacgggg
9	engZR	gcactcacgtgttagaaactagttatttgacctaaaatgtatttttttc
10	pelAhydF	gtttagttccacggggttcaGGCGGGCCGTCCAGCGTG
11	pelAhydR	gtgccatggcatgcgtcgacTCACGGTTGCACCTCGACGTCG
12	411BuilderF	GTCGACGCATGCCATGGC
13	411BuilderR	TGAACCCCGTGGAACTAAACC
14	pSIPseqF	CAGCTCCAGATCTACCGG
15	pSIPseqR	gcaatatcagtaattgctttatcaactgctg
16	engZmidseqF	GAGCTTTGCTTAAAGCACG
17	endAKOF	CTTTCGCTACGTTGCTGGCTCGTTTTAACACGGAGTAAGTGGCTGCTTCGAAGTTCC
18	endAKOR	CTGGCCTTCACCGCCATTCCACTGGCTGTACATAAAGTTGCCTCCTTAGTTCCTATT
19	endAverF	cctgatctggctgattgcatacc
20	endAverR	ctcccagtcggtaaccggatac

Supplementary References

1. Silva, M., Jacobus, N. V., Deneke, C. & Gorbach, S. L. Antimicrobial substance from a human Lactobacillus strain. *Antimicrob. Agents Chemother.* (1987). doi:10.1128/AAC.31.8.1231
2. Kleerebezem, M. *et al.* Complete genome sequence of Lactobacillus plantarum WCFS1. *Proc. Natl. Acad. Sci. U. S. A.* **100**, 1990–1995 (2003).
3. Wiehlmann, L. *et al.* Population structure of Pseudomonas aeruginosa. *Pnas* **104**, 8101–8106 (2007).
4. Lutz, R. & Bujard, H. Independent and tight regulation of transcriptional units in escherichia coli via the LacR/O, the TetR/O and AraC/I1-I2 regulatory elements. *Nucleic Acids Res.* (1997). doi:10.1093/nar/25.6.1203
5. Tjåland, R. Secretion and anchoring of Mycobacterium tuberculosis antigens in Lactobacillus plantarum. (Norwegian University of Life Sciences, 2011).
6. Sørvig, E., Mathiesen, G., Naterstad, K., Eijsink, V. G. H. & Axelsson, L. High-level, inducible gene expression in Lactobacillus sakei and Lactobacillus plantarum using versatile expression vectors. *Microbiology* **151**, 2439–2449 (2005).
